# Kinetics of Viremia and NS1 Antigenemia Are Shaped by Immune Status and Virus Serotype in Adults with Dengue

**DOI:** 10.1371/journal.pntd.0001309

**Published:** 2011-09-06

**Authors:** Vianney Tricou, Nguyet Nguyen Minh, Jeremy Farrar, Hien Tinh Tran, Cameron P. Simmons

**Affiliations:** 1 Oxford University Clinical Research Unit, Wellcome Trust Major Overseas Program, Ho Chi Minh City, Viet Nam; 2 Hospital for Tropical Diseases, Ho Chi Minh City, Viet Nam; 3 Centre for Tropical Medicine, University of Oxford, Oxford, United Kingdom; University of California, Berkeley, United States of America

## Abstract

**Background:**

Dengue is a major public health problem in tropical and subtropical countries. Exploring the relationships between virological features of infection with patient immune status and outcome may help to identify predictors of disease severity and enable rational therapeutic strategies.

**Methods:**

Clinical features, antibody responses and virological markers were characterized in Vietnamese adults participating in a randomised controlled treatment trial of chloroquine.

**Results:**

Of the 248 patients with laboratory-confirmed dengue and defined serological and clinical classifications 29 (11.7%) had primary DF, 150 (60.5%) had secondary DF, 4 (1.6%) had primary DHF and 65 (26.2%) had secondary DHF. DENV-1 was the commonest serotype (57.3%), then DENV-2 (20.6%), DENV-3 (15.7%) and DENV-4 (2.8%). DHF was associated with secondary infection (Odds ratio = 3.13, 95% CI 1.04–12.75). DENV-1 infections resulted in significantly higher viremia levels than DENV-2 infections. Early viremia levels were higher in DENV-1 patients with DHF than with DF, even if the peak viremia level was often not observed because it occurred prior to enrolment. Peak viremias were significantly less often observed during secondary infections than primary for all disease severity grades (*P* = 0.001). The clearance of DENV viremia and NS1 antigenemia occurs earlier and faster in patients with secondary dengue (*P*<0.0001). The maximum daily rate of viremia clearance was significantly higher in patients with secondary infections than primary (*P*<0.00001).

**Conclusions:**

Collectively, our findings suggest that the early magnitude of viremia is positively associated with disease severity. The clearance of DENV is associated with immune status, and there are serotype dependent differences in infection kinetics. These findings are relevant for the rational design of randomized controlled trials of therapeutic interventions, especially antivirals.

## Author Summary

Dengue is an acute viral disease that affects tens of millions of people annually in tropical and sub-tropical countries. In some cases, this infection happens to be severe and even life threatening. Severe cases have been associated with higher levels of virus in the blood. Several hypotheses have been proposed to explain the occurrence of these cases notably by involving the patient's history of previous DEN virus infection(s). Little is known about the relationships between the evolution over time of virus levels in the blood, the clinical outcome and the previous infection(s) history—a better understanding of these features could help in anti-viral drug development. To analyze these relationships, we studied well characterized patients who participated in a clinical trial. The majority of these patients were infected by DENV-1 serotype and had higher levels of virus than those infected by DENV-2 and sometimes DENV-3 serotypes. We also found that patients with more severe symptoms had higher levels of virus in the first days of their illness. We found as well that the virus was cleared faster and earlier from the blood of patients previously infected. These findings are of major importance for further anti-viral drug testing.

## Introduction

Dengue viruses (DENVs) are members of the Flavivirus genus and are the most important arboviral pathogens of humans. The four DENVs are antigenically-related and have single-stranded, positive-sense RNA genomes that share 60–70% sequence identity between each others [1]. There are no licensed vaccines to prevent dengue and vector control remains the cornerstone of public health interventions.

The clinical outcome from DENV infection ranges from the asymptomatic to an acute, often debilitating illness called dengue fever (DF), to the severe and potentially life-threatening dengue hemorrhagic fever (DHF) and dengue shock syndrome (DSS). The cardinal feature of DHF/DSS is a capillary permeability syndrome characterised by plasma leaking from the vasculature into interstitial spaces. Thrombocytopenia, a coagulopathy and a hemorrhagic diathesis are also common findings. DSS manifests when capillary permeability is severe enough to result in an inadequate intravascular volume that then leads to poor tissue perfusion. DSS is managed, and possibly prevented, by careful restoration and maintenance of the intravascular volume by use of parenteral fluids. Viral strain and host immune status have been suggested as major risk factors for DHF/DSS. In particular, two sequential infections, with the second infection caused by a DENV serotype different from the first, is a risk factor for severe disease in children and adults [2]–[5]. A process called antibody dependent enhancement of infection (ADE), coupled with strong anamnestic cellular immune responses is the leading hypothesis to mechanistically explain more severe disease in secondary infections [6]–[9]. Severe dengue can also occur in primary infection of infants born to dengue-immune mothers with, indicating anamnestic immune responses are not absolutely critical for eliciting the capillary permeability syndrome in all patients. Viral traits may also be important in pathogenesis, with strong evidence that some viral genotypes are fitter than others [10], [11].

The literature describing the overall relationship between plasma/serum viral burden, disease severity and immune status generally supports the hypothesis that there is a positive correlation between markers of viral burden in the first 2–3 days of fever and the severity of clinical outcomes. For example, during the febrile and early convalescent periods, Taiwanese adults with secondary DENV-3 infections and DHF had higher plasma levels of viral RNA than did patients with DF [12]. An association between higher peak viremia and increased disease severity was observed in Thai children with acute DENV-1 and -2 infections [13]. Similarly, DHF was associated with higher plasma viremia early in illness in Thai children with secondary DENV-3 infections [14]. Duyen et al recently showed that DENV-1 infections were associated with higher viremia and NS1 antigenemia than DENV-2 infections in ambulatory Vietnamese paediatric patients [15]. In the same patients, viremia and NS1 antigenemia persisted for longer in patients with primary infections.

In adults, where the risk of clinically apparent disease occurring in primary infection is possibly greater [16], [17], there is less evidence relating virological features of infection to immune status or clinical outcome. Kuberski et al reported in 1977 that the magnitude of viremia in young adult patients was higher in primary than secondary DENV-1 infections [18]. More recently, DENV viremia levels from Taiwanese adult patients were reported to be lower in secondary than in primary DENV-2 infections [19].

The dynamics of virus clearance might also be relevant to clinical outcome. In Thai children, Vaughn and others have shown that the slope of the descending portion of the viremia curve was steeper for patients with secondary infection versus those with primary infection and viremia decreased more quickly for patients with DHF than for patients with DF at defervescence [13]. The accelerated clearance of viremia in secondary infection most likely reflects the contribution of anamnestic humoral and cellular immune responses, which themselves have been implicated in the pathogenesis of capillary leakage. Conversely however, Wang et al suggested clearance of the virus and virus-containing immune complexes was slower in adult DHF patients [20].

A better understanding of the relationship between biomarkers of virus infection, the immune response and disease evolution is critical for the rational use of intervention therapies in dengue, e.g. anti-viral drugs or immune-modulating therapies. To this end, this study describes the kinetics of viremia and NS1 in an intensively investigated cohort of Vietnamese adults with dengue and less than 72 hrs of fever enrolled in a randomized placebo-controlled trial (RCT).

## Methods

### Study setting, participants and ethical considerations

A double blind RCT of chloroquine (CQ) in 307 adults hospitalized for suspected DENV infection was conducted at the Hospital for Tropical Diseases (Ho Chi Minh City, Vietnam) between May 2007 and July 2008. Information on recruitment, inclusion criteria, randomization, treatment and investigations have been published previously [21]. The Scientific and Ethical committee of the HTD and the Oxford Tropical Research Ethical Committee approved the study protocol and all patients gave written informed consent. The trial was registered with the ISRCTN Register (ISRCTN38002730). Herein we describe the clinical and virological features of the 257 patients with laboratory confirmed dengue enrolled into this trial, which hitherto have not been described in detail.

### Dengue diagnostics and detection of DENV RNA and NS1 in plasma

A diagnosis of laboratory confirmed dengue was reached using serological, antigen detection and molecular methods [22]. In brief, RT-PCR detection of DENV RNA in plasma was performed using an internally controlled, serotype-specific, real-time RT-PCR TaqMan assay that has been described previously [23]. RNA extraction from plasma samples was automated (NucliSens easyMAG, BioMerieux, Lyon, France). Results were expressed as cDNA equivalents per mL of plasma. A capture IgM and IgG ELISA (MAC and GAC ELISA) using DENV/JEV antigens and mAb reagents provided by Venture Technologies (Sarawak, Malaysia), was performed as previously described [24]. NS1 was detected by using the NS1 Platelia ELISA assay from BioRad (Hercules, CA) according to the manufacturer's instructions. Samples defined as equivocal in the NS1 Platelia ELISA assay were repeated and if they were still equivocal they were regarded as being negative.

### Host immune status

The interpretation of primary and secondary serological responses was based on the magnitude of IgG ELISA units in early convalescent plasma samples taking into account the illness day. The cut-off in IgG ELISA units for distinguishing primary from secondary dengue by illness day was calibrated using a panel of acute and early convalescent sera from Vietnamese dengue patients that were assayed at the Centre for Vaccine Development, Mahidol University, Bangkok, Thailand using a reference IgM and IgG antibody capture ELISA described previously [25].

### Clinical and laboratory investigations

Clinical history and examination findings were recorded daily into case record forms. An ultrasound was performed in all patients within 24 hrs of defervescence. Venous blood samples were collected at hospital admission, then twice daily (around 9am and 3pm) for a minimum of 5 days after hospital admission and again 10–14 days after discharge from the hospital. A complete blood count, including hematocrit (Hct) and platelet measurements, was performed daily for all patients. Hct measurements were performed more frequently if clinically indicated. The extent of hemoconcentration during symptomatic illness was determined by comparing the maximum Hct recorded during hospitalization with either the value recorded at follow-up when available (191/248 i.e. 77% of the patients) or against a sex- and age-matched population value. Plasma was stored frozen in multiple aliquots at −80°C until use in the real-time RT-PCR and NS1 ELISA. The day of fever onset was self-reported by the patient and was designated illness day 1.

### Case definition

DF and DHF were diagnosed according to 1997 World Health Organization (WHO) classification criteria and was applied to each case after review of study notes [26]. The 1997 definitions were used for this study because at the time of clinical assessment the 2009 WHO Guidelines and revised classification scheme was not available. DF was defined as a laboratory confirmed dengue case with no evidence of capillary permeability as defined for a DHF case. DHF was defined as laboratory confirmed dengue case with thrombocytopenia (<100,000 platelets/mm3), any hemorrhagic manifestation, and evidence of plasma leakage (as denoted by a >20% increase in the Hct from the baseline value or by the presence of pleural or abdominal effusions).

### Analysis and statistics

The data used in this analysis was taken from a randomised controlled treatment trial of dengue. Since the intervention (CQ) had no measurable impact on virological or immunological outcomes, for the purposes of this analysis we did not distinguish between patients in the CQ or placebo arms of the study. All statistical analysis was performed and figures designed using the software R (version 2.10.1). Significance was assigned at *P*<0.05 and were two-sided unless otherwise indicated. Uncertainty was expressed by 95% confidence intervals. The Kruskal-Wallis rank sum test was used for continuous variables and the Fisher's exact test for categorical variables. For the viremia kinetics analysis, when the RT-PCR signal was below the assay limit of detection (defined as the last dilution of standard that gave a specific signal), a value equal to concentration of the last dilution of standard that gave a specific signal divided by 10 was assigned. The maximum viremia level was defined as the highest plasma viremia level measured during illness. The maximum viremia level was considered to be a peak viremia level only in cases in which viremia rose after the enrolment specimen. To compare kinetics of viremia between patients with different serological status, disease severity and serotype, the means of log-transformed viremia measurements made on the same illness day were used as a summary measure of the viremia on that day. To estimate the maximum daily rate of DENV clearance, the slope of the viremia curve was calculated for each illness day as the change in the means of log-transformed viremia measurements made on the same illness day. Only the maximum decreasing daily rate of each patient was used for analysis. Survival analysis using the Kaplan-Meier method and log-rank test was used for all time-to-event outcomes. Time to resolution of viremia or NS1 antigenemia was defined as the time from the start of symptoms until the first of two consecutive plasma samples below the RT-PCR limit of detection or NS1 ELISA negative. The fever clearance time (FCT) was defined as the time from the start of symptoms to the start of the first 48 hours period during which axillary temperature remained below 37.5°C.

## Results

### Characteristics of the study population

Of the 307 adults with suspected dengue enrolled in the CQ RCT between May 2007 and July 2008, 257 had laboratory-confirmed dengue including 248 patients with a defined serological and clinical classification and 9 patients with ambiguous or unknown clinical outcomes or serology (mainly because they left the study prematurely). The characteristics of the study population are summarized in Table 1 (and Table S1). DENV-1 (57.3%) was the commonest serotype detected in this population of patients, then DENV-2 (20.6%), DENV-3 (15.7%) and DENV-4 (2.8%). DHF was significantly associated with secondary infection compared with primary infection (65/215 vs 4/33 i.e. 30.2% vs 12.1%, *P* = 0.04, Odds ratio (OR) = 3.13, 95% CI 1.04–12.75). DHF resulting from secondary infection was more commonly associated with DENV-2 (21/45 (46.7%)) than for other serotypes (DENV-1: 33/124 (26.6%), DENV-3: 10/33 (30.3%) and DENV-4: 1/7 (14.3%) (DENV-2 vs DENV-1, -3 and -4 *P* = 0.02, OR = 2.38, 95% CI 1.14–4.96) (Table S1).

**Table 1:** The characteristics of the study population^a^.

	N (%) or Median (interquartile range)	
Variables	Primary (N = 33)	Secondary (N = 215)
**Age (years)**	19 (17–25)	22 (18–27)
**Male sex**	20 (60.6%)	147 (68.4%)
**Viremic**	30 (90.9%)	209 (97.2%)
**Infecting serotype:**		
**DENV-1**	18 (60.0%)	124 (59.3%)
**DENV-2**	6 (20.0%)	45 (21.5%)
**DENV-3**	6 (20.0%)	33 (15.8%)
**DENV-4**	0 (0.0%)	7 (3.4%)
**NS1 ELISA positive**	28 (84.8%)	186 (86.5%)
**Febrile**	31 (93.9%)	209 (97.2%)
**Time since illness onset (hrs)**	51.5 (43.5–68.0)	49.0 (41.3–55.0)
**Disease severity:**		
**DF**	29 (87.9%)	150 (69.8%)
**DHF**	4 (12.1%)	65 (30.2%)

Treatment allocation by disease severity^b^:	CQ	Placebo	CQ	Placebo
**DF**	19 (65.5%)	10 (34.5%)	78 (52.0%)	72 (48.0%)
**DHF**	2 (50.0%)	2 (50.0%)	27 (41.5%)	38 (58.5%)

a Were omitted from this table 9 patients with ambiguous or unknown clinical outcomes or serology (mainly because they left the study prematurely).

b Percentages given here are within the same group of immune status and disease severity.

### Viremia kinetics in DF and DHF according to serological status

**Figure 1:**
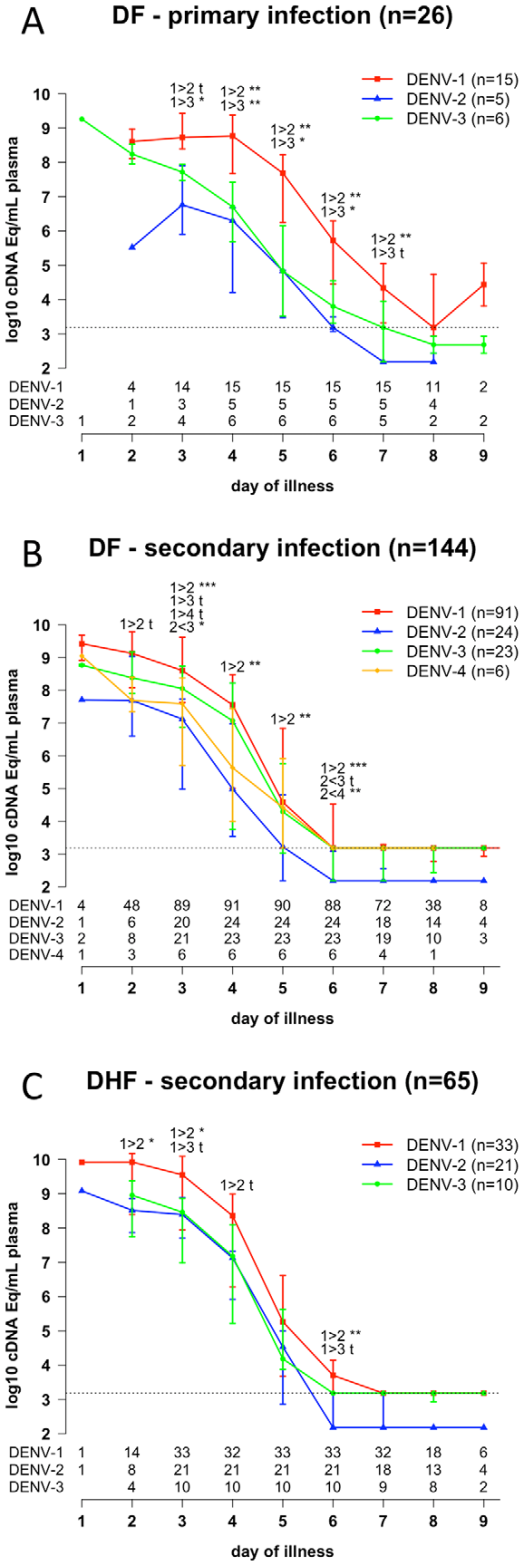
Magnitude of DENV viremia by illness day. Levels of DENV-1, -2, -3 and -4 genome equivalent cDNA copies per millilitre were determined in serial plasma samples from patients with **A**) primary DF (n = 26), **B**) secondary DF (n = 144) and **C**) secondary DHF (n = 64). Data are median and IQR. *** *P*<0.001 ** 0.001<*P*<0.01 * 0.01<*P*<0.05 **t** 0.05<*P*<0.1. Data from patients with DENV-1 and -2 primary DHF (n = 3 and n = 1 respectively) and DENV-4 secondary DHF (n = 1) are not displayed because the number of these patients was too small for statistical analysis. The numbers below the graph indicate the numbers of patients at each time point. The dashed line represents the assay limit of detection.

Median viremia levels by illness day for DENV-1, -2 and -3 are shown in Figure 1 (and Table S2). In DF patients with primary infection, DENV-1 viremia levels were significantly higher than DENV-2 or DENV-3 levels at multiple time-points during the acute illness (Figure 1A). In DF patients with secondary infection, the most common serological and clinical grouping, and DHF patients with secondary infection, DENV-1 levels were significantly higher than DENV-2 levels and there was also a non-significant trend towards higher DENV-1 levels than DENV-3 levels (Figure 1B and C). Collectively, and despite small sample sizes for some subgroups, these data suggest that DENV-1 infections were associated with higher viremias (as measured by qRT-PCR) than DENV-2, irrespective of disease severity and immune status.

**Figure 2:**
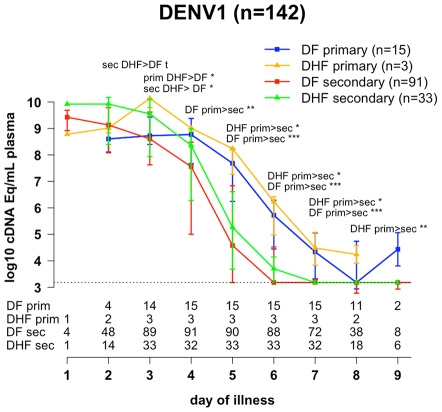
Magnitude of DENV-1 viremia by illness day. Levels of DENV-1 genome equivalent cDNA copies per millilitre were determined in serial plasma samples from DENV-1 infected patients with primary DF (n = 15), primary DHF (n = 3), secondary DF (n = 91) and secondary DHF (n = 33). Data are median and IQR. *** *P*<0.001 ** 0.001<*P*<0.01 * 0.01<*P*<0.05 **t** 0.05<*P*<0.1. The numbers below the graph indicate the numbers of patients at each time point (2 per patient for most of the time points). The dashed line represents the assay limit of detection.

DENV-1 was the commonest serotype detected in this patient population and therefore there was sufficient data to enable direct comparisons of viremia kinetics across serological states and clinical severity whilst controlling for the infecting serotype (Table S2). These data show that in the early acute phase (illness day 3) patients with DENV-1 infection and DHF had significantly higher viremia levels than DENV-1 patients with DF, irrespective of the patient immune status (Figure 2). These data show also that later in the acute phase (from day 4 of illness) patients with primary DENV-1 infections had significantly higher viremia levels than patients with secondary DENV-1 infections, irrespective of the disease severity (Figure 2).

A limitation of these analyses is that in the majority of patients with secondary infections the viremia was already declining at the time of enrolment i.e. we did not observe an obvious peak viremia (Table S3). Overall, a peak viremia was significantly less often observed in secondary infections than in primary infections for all disease severity grades (*P* = 0.001, OR = 3.64, 95% CI 1.55–8.74). However, there were no significant differences in the duration of illness prior to enrolment between patients in different categories of serological status or disease severity (*P* between 0.11 and 0.96 if all the serotypes are considered, and 0.16 and 0.39 if and only DENV-1), suggesting this snapshot of viremia levels is unbiased by differences in duration of illness at study enrolment.

### Timing and amplitude of peak viremia and associations with severity and immune status

Peak viremia levels were identified in 72 patients. Peak viremia occurred significantly earlier in secondary DF than in primary DF (*P* = 0.008) and in secondary DHF than in primary DHF (*P* = 0.04) but there were no significant differences between primary DF and primary DHF (*P* = 0.73) and between secondary DF and secondary DHF (*P* = 0.13) for the peak viremia time (Table S3). Amongst DENV-1 infected patients, peak viremia levels, when observed (in 51 of 142 DENV-1 infected patients), happened significantly earlier in secondary DF than in primary DF (*P* = 0.0006) but, possibly because of small sample size, not in secondary DHF compared to primary DHF (*P* = 0.31). There was no significant difference between secondary DF and secondary DHF (p = 0.48) but there was a non-significant trend towards later viremia peaks in primary DF than in primary DHF (*P* = 0.052).

There were sufficient observations of the magnitude of peak viremia in DENV-1 infections to look for associations with clinical outcome in this subgroup (Table S3). Peaks of viremia were observed in 9 primary DF, 29 secondary DF, 3 primary DHF and 10 secondary DHF DENV-1 infected patients. Peak viremia levels were not significantly different between DF and DHF patients (DF vs DHF log10 median peak levels 9.89 vs 10.27, *P* = 0.28) but there was a non-significant trend towards higher peak viremia during secondary infections than primary infections (primary vs secondary *P* = 0.096 and primary DF vs secondary DF *P* = 0.086). If considering the highest viremia levels (as distinct from peak viremia levels) in DENV-1 patients, these were significantly higher in DHF than in DF (log10 median levels 9.84 vs 9.19, *P* = 0.03). Because most DHF cases were associated with secondary infections, for which peak viremia had already past by the time of enrolment, this difference is probably underestimated. These results suggest secondary infections are generally associated with earlier peak viremia but do not provide any conclusive evidence of higher peak viremia levels in DHF and/or secondary infections.

### Maximum daily rate of virus clearance

In the 239 patients with detectable viremia, the median of maximum daily rates of DENV clearance (estimated as the slope of the steepest descending daily portion of the viremia curve) was 2.2 log10 per day in primary DF, 2.8 log10 per day in secondary DF, 2.1 log10 per day in primary DHF and 3.0 log10 per day in secondary DHF. The maximum daily rate of clearance was significantly higher in patients with secondary infections (median of the maximum daily loss 2.9 logs per day) versus those who experienced primary infections (median of maximum daily losses = 2.1 logs per day, *P*<0.00001) (primary DF vs secondary DF *P* = 0.00004 and primary DHF vs secondary DHF *P* = 0.025). The results were very similar when considering only DENV-1 patients for analysis (data not shown). These data suggest secondary infection is associated with steeper declines in viremia.

### Time to resolution of viremia in DF and DHF according to serological status

**Figure 3:**
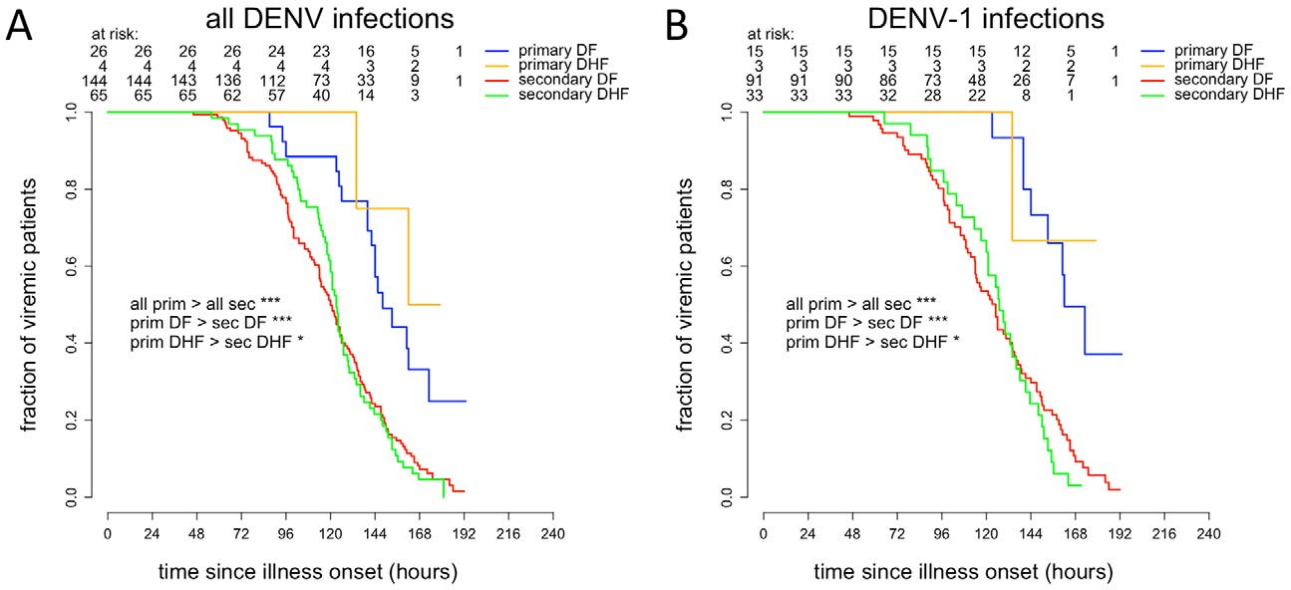
Time to resolution of DENV viremia. Kaplan-Meyer survival analysis of time to resolution of plasma viremia in all viremic **A**) or DENV-1 only **B**) patients.

Amongst all viremic patients (n = 239) time to resolution of viremia was significantly longer in primary infections than in secondary infections (hazard ratio (HR) = 2.88, 95% CI 1.79–4.63, log rank test *P* = 0.000005), in primary DF than in secondary DF (HR = 2.60, 95% CI 1.57–4.32, log rank test *P* = 0.0001) and in primary DHF than in secondary DHF (HR = 4.92, 95% CI 1.19–20.32, log rank test *P* = 0.015) (Figure 3A). Median times to resolution of dengue viremia were 148 hrs (IQR 140–173 hrs) in primary DF, 162 hrs (134–>171 hrs) in primary DHF, 120 hrs (97–141.5 hrs) hrs in secondary DF and 123 hrs (113–138 hrs) in secondary DHF.

Amongst DENV-1 infected patients only (n = 142), times to resolution of viremia were also significantly longer in primary infections than in secondary infections (HR = 4.21, 95% CI 2.12–8.35, log rank test *P* = 0.000009), in primary DF than in secondary DF (HR = 3.67, 95% CI 1.76–7.62, log rank test *P* = 0.0002) and in primary DHF than in secondary DHF (HR = 7.00, 95% CI 0.94–52.17, log rank test *P* = 0.03) (Figure 3B). Median times to resolution of DENV-1 viremia were 162 hrs (IQR 144–>176 hrs) in primary DF, >171.1 hrs (134–>179 hrs) in primary DHF (since less than 50% of primary DHF had cleared viremia before discharge), 125 hrs (99–150 hrs) in secondary DF and 127 hrs (107–143.5) in secondary DHF.

### Time to resolution of NS1 antigenemia in DF and DHF according to serological status

**Figure 4:**
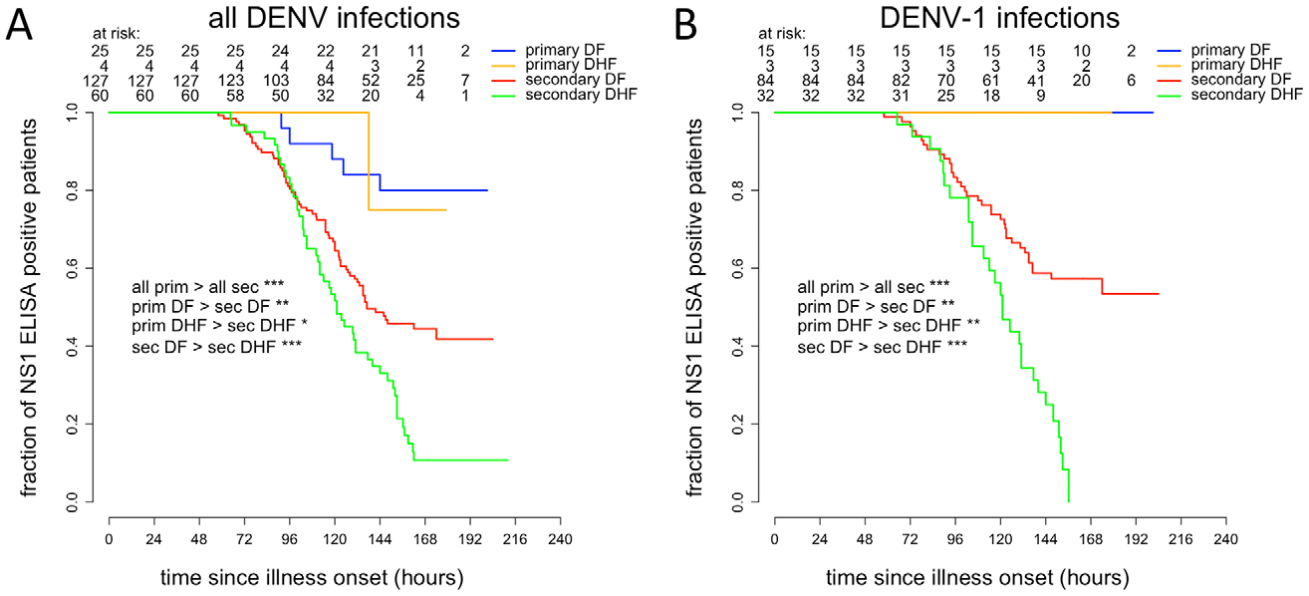
Time to resolution of NS1 antigenemia. Kaplan-Meyer survival analysis of time to resolution of NS1 antigenemia in all viremic **A**) or DENV-1 only **B**) patients. Two patients NS1 negative at enrolment were later positive and for the purposes of analysis were considered positive at the time of enrolment.

Of the 248 patients with defined serological and clinical classifications, there were 214 patients NS1 positive at the time of study enrolment (plus 2 patients negative at enrolment but NS1 positive 24 and 42 hrs later). Consistent with the viremia findings, times to resolution of NS1 antigenemia were significantly longer in primary infections than in secondary infections (HR = 4.57, 95% CI 2.01–10.40, log rank test *P* = 0.00007), in primary DF than in secondary DF (HR = 3.66, 95% CI 1.47–9.07, log rank test *P* = 0.003), in primary DHF than in secondary DHF (HR = 7.04, 95% CI 0.97–51.15, log rank test *P* = 0.02) but also in secondary DF than in secondary DHF (HR = 1.86, 95% CI 1.29–2.67, log rank test *P* = 0.0007) (Figure 4A). Interestingly, only 5 of 25 patients (i.e. 20%) with primary DF and 1 of 4 patients (i.e. 25%) with primary DHF had cleared NS1 when discharged from hospital. In patients with secondary dengue, 69 of 127 with secondary DF (i.e. 54.3%) and 51 of 60 with secondary DHF (i.e. 85%) had cleared NS1 when discharged from hospital. Median times to resolution of NS1 antigenemia since illness onset were >166 hrs (>146–>178 hrs) in primary DF and >158 hrs (138–>171 hrs) in primary DHF (since less than 50% of primary DF and primary DHF had cleared NS1 before discharge), and 137 hrs (105–>174 hrs) in secondary DF and 121 hrs (100–153 hrs) in secondary DHF.

Amongst DENV-1 infected patients (n = 142), 134 were NS1 positive at the time of study enrolment. Times to resolution of NS1 antigenemia were significantly longer in DENV-1 primary infections than in DENV-1 secondary infections (HR = not applicable, log rank test *P* = 0.00008), in DENV-1 primary DF than in DENV-1 secondary DF (HR = 3.66, 95% CI 1.47–9.07, log rank test *P* = 0.003), in DENV-1 primary DHF than in DENV-1 secondary DHF (HR = 7.04, 95% CI 0.97–51.15, log rank test *P* = 0.004) and in DENV-1 secondary DF than in DENV-1 secondary DHF (HR = 1.86, 95% CI 1.29–2.67, log rank test *P* = 0.00001) (Figure 4B). Strikingly, none of the DENV-1 infected patients with primary DF (n = 15) or primary DHF (n = 3) had cleared NS1 when they were discharged from hospital. In contrast, 36 of 84 (42.9%) secondary DF and 29 of 31 (93.5%) secondary DHF patients had cleared NS1 when they were discharged. Median times to resolution of NS1 antigenemia since illness onset were >172.5 hrs in primary DF, >171 hrs in primary DHF, >174 hrs in secondary DF (since less than 50% of primary and secondary DF, and primary DHF had cleared NS1 before discharge) and 121 hrs (103–144 hrs) in secondary DHF.

Collectively, these results suggest that DENV infection is cleared earlier and faster in secondary infections than in primary infections.

### Fever clearance time in DF and DHF according to serological status

**Figure 5:**
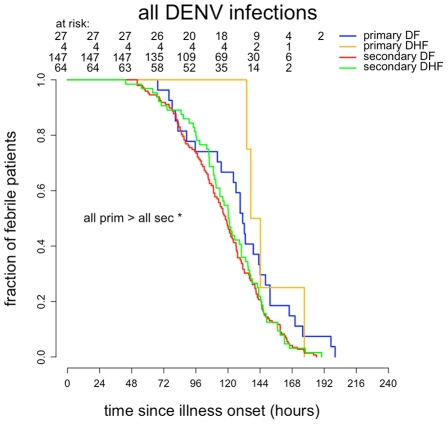
Fever clearance time. Kaplan-Meyer survival analysis of fever clearance time in all viremic patients. Two patients afebrile at enrolment developed fever soon after and for the purposes of analysis were considered febrile at the time of enrolment.

There were 240 patients febrile at enrolment (plus 2 afebrile patients who developed fever soon after). Overall, FCT were significantly longer in primary infections than in secondary infections (log rank test *P* = 0.037) but there was no significant difference between primary DF and secondary DF (HR = 1.44, 95% CI 0.93–2.23, log rank test *P* = 0.096), and between primary DHF and secondary DHF (HR = 1.85, 95% CI 0.67–5.14, log rank test *P* = 0.23) (Figure 5). Median FCT since illness onset was 131 hrs (IQR 95.5–151.4 hrs) in primary DF, 141 hrs (135.5–160.5 hrs) in primary DHF, 118 hrs (93.1–140.2 hrs) in secondary DF and 120 hrs (105–142 hrs) in secondary DHF. Consistent with the viremia and NS1 findings, these data indicate primary infection was associated with a longer-lived febrile period.

### DENV-1 viremia kinetics and associations with haematological parameters

**Figure 6:**
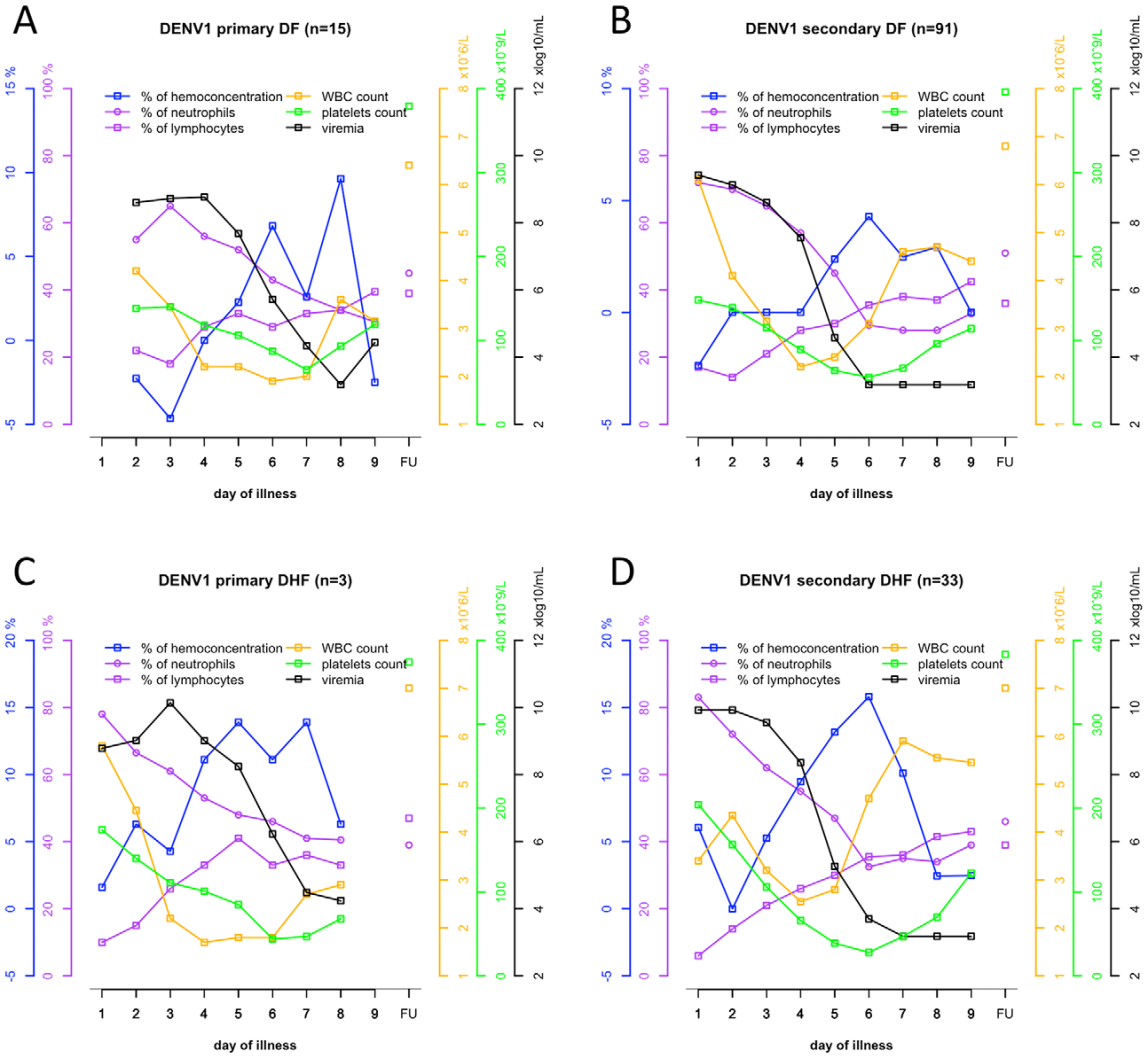
Profile of haematological parameters and viremia in DENV-1 infected patients. Medians of viremia levels, WBC and platelet counts, hemoconcentration, and percentages of lymphocytes and neutrophils were determined in serial plasma samples from DENV-1 infected patients with **A**) primary DF, **B**) secondary DF, **C**) primary DHF and **D**) secondary DHF. The numbers below the graph indicate the numbers of patients at each time point. For reasons of clarity, error bars are not shown.

For descriptive purposes, the evolution over time of DENV-1 viremia in the context of white blood cell and platelet counts and percentage hemoconcentration was plotted (Figure 6) and summarised in Table S4. The highest levels of hemoconcentration and the lowest platelet counts occur when the viremia is close to resolution and when the patient is already or very nearly afebrile. The time to platelet nadir and maximum hemoconcentration was shorter in secondary DENV-1 infections (*P*<0.01). The data also suggest that leucopenia lasts longer in primary infections than in secondary infections.

## Discussion

The interplay between DEN virus infection and host immune status is postulated to play a central role in the pathophysiology of severe dengue. In this current study, we observed important features of this dynamic. First, early viremia levels were higher in patients with DHF, even if the peak viremia level was often not observed because it occurred prior to enrolment. Second, DENV-1 infections manifested as higher and longer-lived viremias, suggesting serotype dependent differences in infection kinetics. Third, the clearance of DENV viremia and NS1 antigenemia occurs earlier and faster in patients with secondary dengue and is also consistent with a faster time to defervescence.

Our findings are in agreement with previous studies that found higher viremias associated with more severe disease [12]–[14]. Our data also suggests that quantitative differences exist between DENV serotypes with respect to the kinetics of viremia and NS1 antigenemia. In particular, DENV-1 infections were associated with higher and frequently longer-lived viremia levels than infections with either DENV-2 or DENV-3. This is in agreement with recent observations in Vietnamese children and adults [15], [27]. DENV-2 was associated with secondary infection and severe disease in our study; this is also in accordance with previous studies [2], [27], [28]. The mechanisms that facilitate relatively higher viremia in DENV-1 infections relative to DENV-2 infections in our study population, and also recently in Vietnamese children [15], are unknown. Plausibly, DENV-1 has an intrinsically faster rate of replication in this patient population and thereby attains a higher infected cell mass in vivo than DENV-2. Clearly, further studies will be needed to explore this.

The duration of NS1 antigenemia was shorter in patients with secondary infections and this is consistent with previous studies that have suggested reduced sensitivity of NS1-based diagnostic tests in patients with secondary infections [22], [29]–[32]. One explanation is that NS1 is less likely to be available for detection when a sufficient level of DENV-reactive IgG (including anti-NS1 IgG) develops during secondary infections. This may serve to mask the antigen from detection in the immunoassay, and/or result in rapid clearance of NS1 in the form of immune-complexes.

Secondary dengue is associated with faster resolution of viremia infection and shorter duration of fever. Interestingly, the daily rates of virus clearance observed in our study were very compatible with those found by Vaughn et al in Thai children [13]. The early adaptive immune response during secondary infection is dominated by populations of memory B and T cells (and possibly memory-like NK cells [33]) and at least some components of this response mediate a strong anti-viral action, as evidence by faster clearance rates of the viremia and the NS1 antigenemia. Clearly however, aspects of this rapid host immune response are clinically deleterious given the epidemiological association between secondary dengue and more severe outcomes, and also the timing of when clinical manifestations of capillary permeability occur. This poses the intriguing question of whether modulating the host immune response (e.g. through early corticosteroid therapy) could achieve both a more gradual clearance of the virus and a host immune response that elicits less pathology.

Assuming blood viremia is a reasonable surrogate of the whole-body virus burden, then the rapid decline of viremia 48–72 hrs after illness onset, especially in secondary infections that carry higher risk for severe outcomes, has implications for rational design of therapeutic pharmacological interventions. An efficacious anti-viral will need favourable pharmacokinetic properties and potency if it is to impact on the viral burden in a rapid and clinically significant manner. Pharmacological targeting (e.g. with corticosteroids) of the host immune response, which accounts for the rapid decline of viremia but which also likely contributes to the capillary permeability syndrome, may equally need to be administered early on in illness e.g. in a prophylactic way, to prevent clinical complications such as DSS. The rapid decline in viremia in secondary infections also highlights the importance of early diagnosis, since early diagnosis will provide the greatest opportunity for an intervention (e.g. an anti-viral), to have an impact. Point of care NS1 diagnostics are available but more can be done to improve their sensitivity [34]. More clinical research is also needed to understand if the sensitivity and specificity of early clinical diagnoses (and prognosis) can be improved, particularly in primary health care settings. Recent literature suggests this is feasible [35], [36].

Current animal models of DENV infection are able to provide for in vivo measurements of anti-viral activity [37], [38]. However these models do not reproduce the temporal changes in virological biomarkers and clinical manifestations seen in naturally infected dengue patients. The lack of concordance between virological and clinical events in small animal models, and what occurs in patients, needs to be carefully considered when evaluating candidate anti-viral drugs for dengue. For instance, the onset of vascular permeability does not follow the disappearance of virus during enhanced DENV infection in mouse models [38], [39].

There are several limitations to our study. Our results are derived from hospitalized patients who are not necessarily representative of patients being seen at the primary health care level. Interestingly however, the same themes identified in this study in hospitalized Vietnamese adults were also observed in Vietnamese children presenting to primary health care level clinics in Ho Chi Minh City [15]. Of the 248 patients in this study with a defined serological and clinical classification, 126 received a 3 day course of CQ and, whilst no virological or immunological effects were detected, an effect of CQ on the clinical phenotype cannot be excluded because vomiting was more frequent in this treatment arm, possibly leading to more dehydration [21]. The majority of the patients in this study were infected by DENV-1. Very few had primary DHF (4 of 248). RT-PCR measurements of viremia in plasma may not be an entirely accurate surrogate of the infected cell mass in vivo, although it is certainly a better surrogate than NS1 antigenemia, which persists well after the febrile period and is heavily influenced by the immune status of the host (i.e. primary versus secondary). Viremia measurements assessed by RT-PCR encompass both infectious and non-infectious viral particles, and the relative proportions may vary between the different serological responses and/or serotypes [40]. Assessment of plasma virus titers based on plaque titration would have been a valid alternative approach to measuring virus concentrations in plasma, however this biological assay is more difficult to standardize and validate relative to a RT-PCR assay. Another general limitation of measuring virus concentrations in plasma is that viruses might be sequestered in other tissues but inaccessible to measurement while still playing a role in disease pathogenesis.

Our study emphasizes the importance of the period before and just after the onset of fever. This and other studies have established that early viremia levels are associated with disease severity, although they are very clearly not the only determinant of outcome. Very little is known of the virological events in the hours preceding and shortly after fever onset, mainly because this is very difficult to investigate without a good experimental model. An interesting insight was provided by clinical trials of DENV-1, -3 and -4 monovalent live attenuated vaccines in the 1980s [41]–[43]. These vaccines were not sufficiently attenuated and some volunteers developed dengue fever. These studies suggest that viremic period starts several days before the onset of symptoms This presymptomatic viremic period should not be underestimated because of its possible contribution to DENV transmission to uninfected mosquitoes. In our study, we also observed prolonged times of virus clearance in primary dengue, and long-lived higher viremia levels in DENV-1 infections. These might lead to a higher possibility of human to mosquito virus transmission by maintaining viremia above the infectious level over a longer period of time.

Collectively, our findings reveal important patterns of the viremia and NS1 antigenemia kinetics according to the patient immune response, disease severity and virus serotype, and may help for the rational design of clinical trials of therapeutic interventions, especially antivirals.

## Supporting Information

Table S1 **The characteristics of the study population by serotype.**
(DOC)

Table S2 **Levels of viremia among the study population by illness day and serotype.**
(DOC)

Table S3 **Peak viremia analysis.**
(DOC)

Table S4 **Maximum of hemoconcentration, platelet and WBC nadirs and their occurrence times by disease severity.** All these markers were determined in serial plasma samples from DENV-1 infected patients with primary DF (n = 15), primary DHF (n = 3), secondary DF (n = 91) and secondary DHF (n = 33).
(DOC)
