# Supplementary material for: Kinetics of Viremia and NS1 Antigenemia Are Shaped by Immune Status and Virus Serotype in Adults with Dengue
Source: PLoS Negl Trop Dis. 2011 Sep 6;5(9):e1309. doi: 10.1371/journal.pntd.0001309 (PMC3167785; doi:10.1371/journal.pntd.0001309)
Supplement: Table S1 — The characteristics of the study population by serotype. (DOC) [file pntd.0001309.s001.doc]

|  | **N (%) or Median (interquartile range)** | | | |
| --- | --- | --- | --- | --- |
| **Variables** | **Primary DF** | **Secondary DF** | **Primary DHF** | **Secondary DHF** |
|  | **DENV-1 (N=142)** | | | |
| **Number of patients** | **15 (10.6%)** | **91 (64.1%)** | **3 (2.1%)** | **33 (23.2%)** |
| **Age (years)** | 18 (17-21) | 21 (18-26) | 18 (17-20) | 21 (17-28) |
| **Male sex** | 8 (53.3%) | 65 (71.4%) | 0 (0.0%) | 20 (60.6%) |
| **NS1 ELISA positive** | 15 (100%) | 84 (92.3%) | 3 (100%) | 32 (97.0%) |
| **Febrile** | 15 (100%) | 89 (97.8%) | 3 (100%) | 32 (97.0%) |
| **Time since illness onset (hrs)** | 51.5 (45.8-56.5) | 46.0 (40.0-53.3) | 33.0 (23.5-46.5) | 51.0 (39.5-55.0) |
|  | **DENV-2 (N=51)** | | | |
| **Number of patients** | **5 (9.8%)** | **24 (47.1%)** | **1 (2.0%)** | **21 (41.2%)** |
| **Age (years)** | 26 (25-28) | 20 (17-25) | 15 | 25 (21-31) |
| **Male sex** | 5 (100%) | 14 (58.3%) | 1 (100%) | 12 (57.1%) |
| **NS1 ELISA positive** | 3 (60.0%) | 14 (58.3%) | 1 (100%) | 16 (76.2%) |
| **Febrile** | 5 (100%) | 24 (100%) | 1 (100%) | 21 (100%) |
| **Time since illness onset (hrs)** | 70.0 (48.0-72.0) | 52.5 (47.5-60.4) | 72 | 53.5 (47.5-63.0) |
|  | **DENV-3 (N=39)** | | | |
| **Number of patients** | **6 (15.4%)** | **23 (59.0%)** | **-** | **10 (25.6%)** |
| **Age (years)** | 22 (18-28) | 21 (18-27) | - | 19 (17-24) |
| **Male sex** | 4 (66.7%) | 14 (60.9%) | - | 8 (80.0%) |
| **NS1 ELISA positive** | 5 (83.3%) | 21 (91.3%) | - | 10 (100%) |
| **Febrile** | 5 (83.3%) | 22 (95.7%) | - | 10 (100%) |
| **Time since illness onset (hrs)** | 48.8 (34.1-66.4) | 50.5 (44.5-53.0) | - | 50.5 (46.0-56.6) |
|  | **DENV-4 (N=7)** | | | |
|  | **-** | **6 (85.7%)** | **-** | **1 (14.3%)** |
| **Age (years)** | - | 26 (23-28) | - | 33 |
| **Male sex** | - | 6 (100%) | - | 1 (100%) |
| **NS1 ELISA positive** | - | 6 (100%) | - | 1 (100%) |
| **Febrile** | - | 6 (100%) | - | 1 (100%) |
| **Time since illness onset (hrs)** | - | 48.0 (39.0-53.3) | - | 57.0 |
