# Supplementary material for: Kinetics of Viremia and NS1 Antigenemia Are Shaped by Immune Status and Virus Serotype in Adults with Dengue
Source: PLoS Negl Trop Dis. 2011 Sep 6;5(9):e1309. doi: 10.1371/journal.pntd.0001309 (PMC3167785; doi:10.1371/journal.pntd.0001309)
Supplement: Table S2 — Levels of viremia among the study population by illness day and serotype. (DOC) [file pntd.0001309.s002.doc]

| **Illness day** | **1** | **2** | **3** | **4** | **5** | **6** | **7** | **8** | **9** |
| --- | --- | --- | --- | --- | --- | --- | --- | --- | --- |
|  | **Viremia (log10 cDNA Eq / mL of plasma) - median (interquartile range)** | | | | | | | | |
|  | **DENV-1 (N=142)** | | | | | | | | |
| **DF primary** | NA | 8,61 (8,11-8,97) | 8,73 (8,40-9,43) | 8,77 (7,67-9,38) | 7,69 (6,24-8,23) | 5,72 (4,45-6,29) | 4,34 (3,32-5,05) | 3,18 (2,94-4,74) | 4,44 (3,81-5,06) |
| **DF secondary** | 9,43 (8,92-9,69) | 9,13 (8,08-9,79) | 8,61 (7,63-9,62) | 7,56 (5,01-8,48) | 4,58 (3,18-6,84) | 3,18 (3,18-4,52) | 3,18 (3,18-3,29) | 3,18 (2,78-3,18) | 3,18 (2,93-3,18) |
| **DHF primary** | 8,78 | 9,02 (8,83-9,20) | 10,14 (9,79-10,14) | 9,01 (8,89-9,01) | 8,24 (7,27-8,25) | 6,23 (4,98-6,42) | 4,49 (3,83-5,08) | 4,24 (3,91-4,58) | NA |
| **DHF secondary** | 9,92 (9,92-9,92) | 9,92 (8,40-10,17) | 9,55 (7,94-10,09) | 8,36 (6,28-8,99) | 5,27 (3,68-6,61) | 3,70 (3,18-4,15) | 3,18 (3,18-3,18) | 3,18 (3,18-3,18) | 3,18 (3,18-3,18) |
|  | **DENV-2 (N=51)** | | | | | | | | |
| **DF primary** | NA | 5,52 | 6,76 (5,90-7,90) | 6,30 (4,21-6,67) | 4,84 (3,48-4,86) | 3,18 (3,07-3,49) | 2,18 (2,18-2,18) | 2,18 (2,18-2,43) | NA |
| **DF secondary** | 7,71 | 7,69 (6,61-9,04) | 7,12 (4,99-7,73) | 4,99 (3,54-6,98) | 3,22 (2,18-4,81) | 2,18 (2,18-3,08) | 2,18 (2,18-2,56) | 2,18 (2,18-2,18) | 2,18 (2,18-2,18) |
| **DHF primary** | NA | NA | NA | 8,005 | 7,99 | 5,855 | 3,76 | 3,18 | NA |
| **DHF secondary** | 9,08 | 8,52 (7,87-8,86) | 8,39 (7,71-8,88) | 7,13 (5,92-7,33) | 4,53 (2,86-5,00) | 2,18 (2,18-3,18) | 2,18 (2,18-3,18) | 2,18 (2,18-2,18) | 2,18 (2,18-2,18) |
|  | **DENV-3 (N=39)** | | | | | | | | |
| **DF primary** | 9,26 | 8,24 (7,95-8,53) | 7,72 (7,47-7,94) | 6,70 (5,69-7,42) | 4,83 (3,52-6,15) | 3,80 (3,31-4,55) | 3,18 (2,18-3,95) | 2,68 (2,43-2,93) | 2,68 (2,43-2,93) |
| **DF secondary** | 8,77 (8,75-8,80) | 8,38 (7,90-9,17) | 8,06 (6,88-8,74) | 7,07 (3,75-8,22) | 4,29 (3,02-5,76) | 3,18 (2,18-3,18) | 3,18 (2,18-3,18) | 3,18 (2,43-3,18) | 3,18 (3,18-3,18) |
| **DHF primary** | NA | NA | NA | NA | NA | NA | NA | NA | NA |
| **DHF secondary** | NA | 8,96 (7,75-9,37) | 8,46 (6,98-8,87) | 7,18 (5,22-8,09) | 4,18 (3,88-5,63) | 3,18 (3,18-3,18) | 3,18 (3,18-3,18) | 3,18 (2,93-3,18) | 3,18 (3,18-3,18) |
|  | **DENV-4 (N=7)** | | | | | | | | |
| **DF primary** | NA | NA | NA | NA | NA | NA | NA | NA | NA |
| **DF secondary** | 9,05 | 7,69 (7,34-8,40) | 7,59 (5,71-8,38) | 5,64 (4,00-7,44) | 4,42 (3,18-5,91) | 3,18 (3,18-3,18) | 3,18 (3,18-3,18) | 3,18 | NA |
| **DHF primary** | NA | NA | NA | NA | NA | NA | NA | NA | NA |
| **DHF secondary** | NA | NA | 8,4 | 7,985 | 6,52 | 3,18 | 3,18 | 3,18 | NA |
