# Supplementary material for: Kinetics of Viremia and NS1 Antigenemia Are Shaped by Immune Status and Virus Serotype in Adults with Dengue
Source: PLoS Negl Trop Dis. 2011 Sep 6;5(9):e1309. doi: 10.1371/journal.pntd.0001309 (PMC3167785; doi:10.1371/journal.pntd.0001309)
Supplement: Table S3 — Peak viremia analysis. (DOC) [file pntd.0001309.s003.doc]

|  | **N (%) or Median (interquartile range)** | | | |
| --- | --- | --- | --- | --- |
| **Variables** | **DF primary** | **DF secondary** | **DHF primary** | **DHF secondary** |
|  | **All serotypes (N=239)** | | | |
| Number of patients | 26 | 144 | 4 | 65 |
| Time since illness onset (days) | 2.12 (1.91-2.74) | 2.00 (1.71-2.29) | 1.94 (1.18-2.63) | 2.08 (1.73-2.29) |
| Number of patients with peak | 13 (50%) | 37 (25.7%) | 4 (100%) | 18 (27.7%) |
| Time of peak (days) | 3.00 (2.42-3.19) | 2.46 (2.13-2.78) | 2.65 (2.53-3.03) | 2.21 (1.99-2.53) |
|  | **DENV-1 (N=142)** | | | |
| Number of patients | 15 | 91 | 3 | 33 |
| Highest viremia (log10 copies / mL of plasma) | 9.58 (8.91-9.76) | 9.14 (8.11-9.81) | 10.26 (9.88-10.27) | 9.82 (8.33-10.26) |
| Time since illness onset (days) | 2.15 (1.91 – 2.36) | 1.92 (1.67-2.22) | 1.38 (0.98-1.94) | 2.13 (1.65-2.29) |
| Number of patients with peak | 9 (60%) | 29 (23.1%) | 3 (100%) | 10 (30.3%) |
| Time of peak (days) | 3.08 (2.98-3.25) | 2.50 (2.25 -2.77) | 2.58 (2.48-2.65) | 2.38 (2.01-2.57) |
| Peak viremia (log10 copies / mL of plasma) | 9.67 (9.02-9.69) | 10.11 (9.41-10.38) | 10.26 (9.88-10.27) | 10.33 (9.99-10.40) |
