# Supplementary material for: Kinetics of Viremia and NS1 Antigenemia Are Shaped by Immune Status and Virus Serotype in Adults with Dengue
Source: PLoS Negl Trop Dis. 2011 Sep 6;5(9):e1309. doi: 10.1371/journal.pntd.0001309 (PMC3167785; doi:10.1371/journal.pntd.0001309)
Supplement: Table S4 — Maximum of hemoconcentration, platelet and WBC nadirs and their occurrence times by disease severity. All these markers were determined in serial plasma samples from DENV-1 infected patients with primary DF (n = 15), primary DHF (n = 3), secondary DF (n = 91) and secondary DHF (n = 33). (DOC) [file pntd.0001309.s004.doc]

|  | **Median (interquartile range)** | | | |
| --- | --- | --- | --- | --- |
| **Variables** | **DF primary** | **DF secondary** | **DHF primary** | **DHF secondary** |
| Maximum of hemoconcentration (%) | 11,6 (6,9-14,2) | 7,7 (4,3-13,4) | 23,0 (21,7-26,8) | 21,1 (14,6-26,5) |
| Illness day at maximum of hemoconcentration | 7 (6-7) | 6 (4-7) | 4 (4-5) | 5 (4-6) |
| Platelet nadir (x1000/mm3) | 61 (49-81) | 46 (33-60) | 44 (42-45) | 25 (17-33) |
| Illness day at platelet nadir | 7 (7-7) | 6 (5-6) | 6 (6-7) | 6 (5-7) |
| WBC nadir (x1000/mm3) | 1,9 (1,2-2,0) | 1,9 (1,5-2,5) | 1,7 (1,6-1,8) | 2,2 (1,8-2,8) |
| Illness day at WBC nadir | 5 (4-6) | 4 (4-5) | 4 (4-5) | 4 (3-4) |
